# The role of continuous renal replacement therapy in the management of cardiorenal syndrome involving acute myocardial infarction with concomitant pneumonia: case report

**DOI:** 10.11604/pamj.2024.49.30.45195

**Published:** 2024-10-09

**Authors:** Resiana Karnina, Vera Irawany, Sidharta Kusuma Manggala, Justika Usmadhani Aulya, Muhammad Faruk

**Affiliations:** 1 Department of Anesthesiology and Intensive Care, Faculty of Medicine, Universitas Indonesia, Dr Cipto Mangunkusumo Hospital, Jakarta, Indonesia,; 2 Department of Anesthesiology and Intensive Care, Faculty of Medicine, Universitas Muhammadiyah Jakarta, Jakarta, Indonesia,; 3 Department of Anesthesiology and Intensive Care, Universitas Indonesia, Fatmawati Hospital, Jakarta, Indonesia,; 4 Tugu Koja Hospital, Jakarta, Indonesia,; 5 Department of Surgery, Faculty of Medicine, Hasanuddin University, Hasanuddin University Hospital, Makassar, Indonesia

**Keywords:** Heart failure, cardiorenal syndrome, septic shock, ultrafiltration, case report

## Abstract

Acute heart failure is associated with high hospitalization and mortality rates. A strong, independent risk factor for mortality in patients with heart failure is acute kidney injury, and the condition caused by this connection between disturbances in heart function and proper kidney functioning is cardiorenal syndrome (CRS). This case report discusses the role of continuous renal replacement therapy (CRRT) in the management of a CRS case with septic shock due to pneumonia. A 56-year-old female patient with a history of acute heart failure developed complications of acute kidney dysfunction and was diagnosed with CRS type 1. Standard management was conducted in the intensive cardiac care unit, but the condition of the patient worsened. The patient was admitted to the intensive care unit and underwent CRRT, after which the kidney function and hemodynamic performance of the patient improved. Therefore, the use of CRRT can be a therapeutic option for CRS patients. CRRT acts as an ultrafiltration mechanism that removes circulating cytokines from the blood, reduces volume overload, and addresses electrolyte imbalance, thus enhancing the functioning of the heart and kidneys and potentially improving prognoses.

## Introduction

Cardiorenal syndrome (CRS) is a disorder affecting both the heart and kidneys [1]. CRS type 1 manifests in approximately 25% of the patients who are admitted to hospital for acute decompensated heart failure (ADHF). Conservative therapy methods are often inadequate for CRS patients due to ongoing kidney deterioration. Ultrafiltration is an alternative to diuretic therapy for patients with refractory kidney failure, and continuous renal replacement therapy (CRRT) is a potential modality [2,3]. This case report discusses the role of continuous renal replacement therapy (CRRT) in the management of a CRS case with septic shock due to pneumonia.

## Patient and observation

**Patient information:** a 56-year-old female patient diagnosed with ADHF due to acute myocardial infarction (AMI) had been hospitalized in the intensive cardiac care unit (ICCU) for 16 days. Suddenly, the patient complained of worsening shortness of breath. Revascularization was not performed due to a family disagreement. The patient had a history of stroke infarction one year ago.

**Clinical findings:** the vital signs of the patient were as follows: GCS E3M4V6, blood pressure 131/68 mmHg, heart rate 71 bpm on dobutamine 5 mcg, respiratory rate 34 breaths/minute. A physical examination revealed rhonchi in both lung fields. During intensive cardiac care, the urine output of the patient decreased and a positive cumulative fluid balance was observed.

**Diagnostic assessment:** the laboratory tests revealed the following: Hb 9.7 g/dL, leukocytes 16,800/mm^3^, arterial blood gas analysis indicating respiratory alkalosis (pH 7.56, pCO2 21.9 mmHg, pO2 219.2 mmHg, HCO3 19.8 mEq/L), and procalcitonin 2.93 ng/mL. The echocardiogram showed an ejection fraction (EF) of 33% with a hypokinetic left ventricle. The chest X-ray revealed bilateral infiltrates, left pleural effusion, and cardiomegaly.

**Therapeutic interventions:** the patient was transferred to the intensive care unit (ICU), intubated, and placed on mechanical ventilation to reduce the breathing effort. Resuscitation with inotropic support and broad-spectrum antibiotics was initiated due to a suspected bacterial infection. A decline in kidney function (urea 228.2 mg/dL, creatinine 3.25 mg/dL, and eGFR 16 mL/min) led to a diagnosis of CRS type 1 with severe septic shock due to pneumonia, and supportive hemodialysis (HD) was performed. On the first day of HD, which lasted 2.5 hours (UF 1000), the hemodynamics of the patient were unstable (blood pressure 126/65 mmHg, heart rate 58 bpm, respiratory rate 32 breaths/minute), and arrhythmia occurred, leading to the discontinuation of HD and the initiation of CRRT.

**Figure 1:**
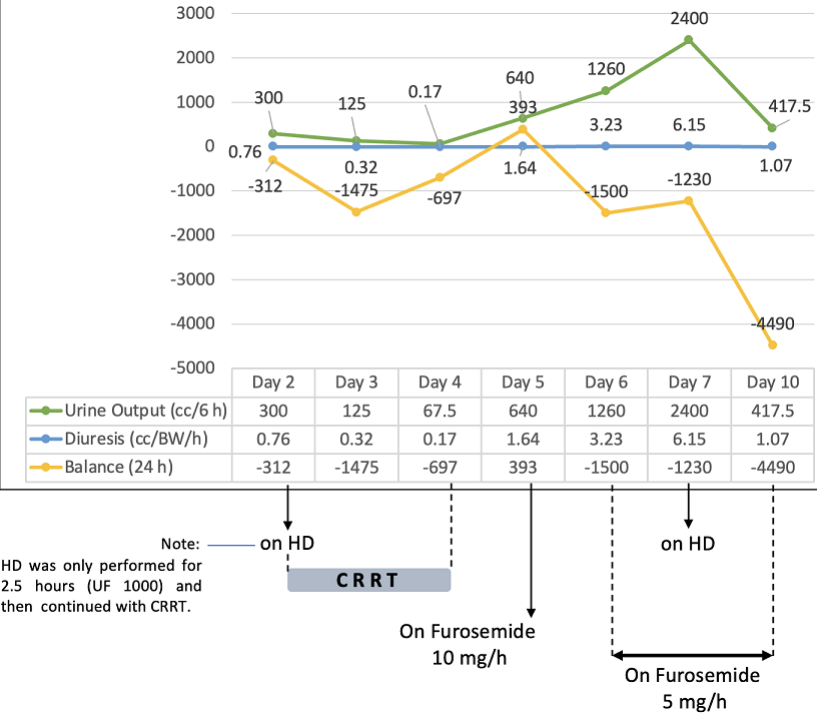
urine output, diuresis, and balance development of the case patient during ICU admission

**Figure 2:**
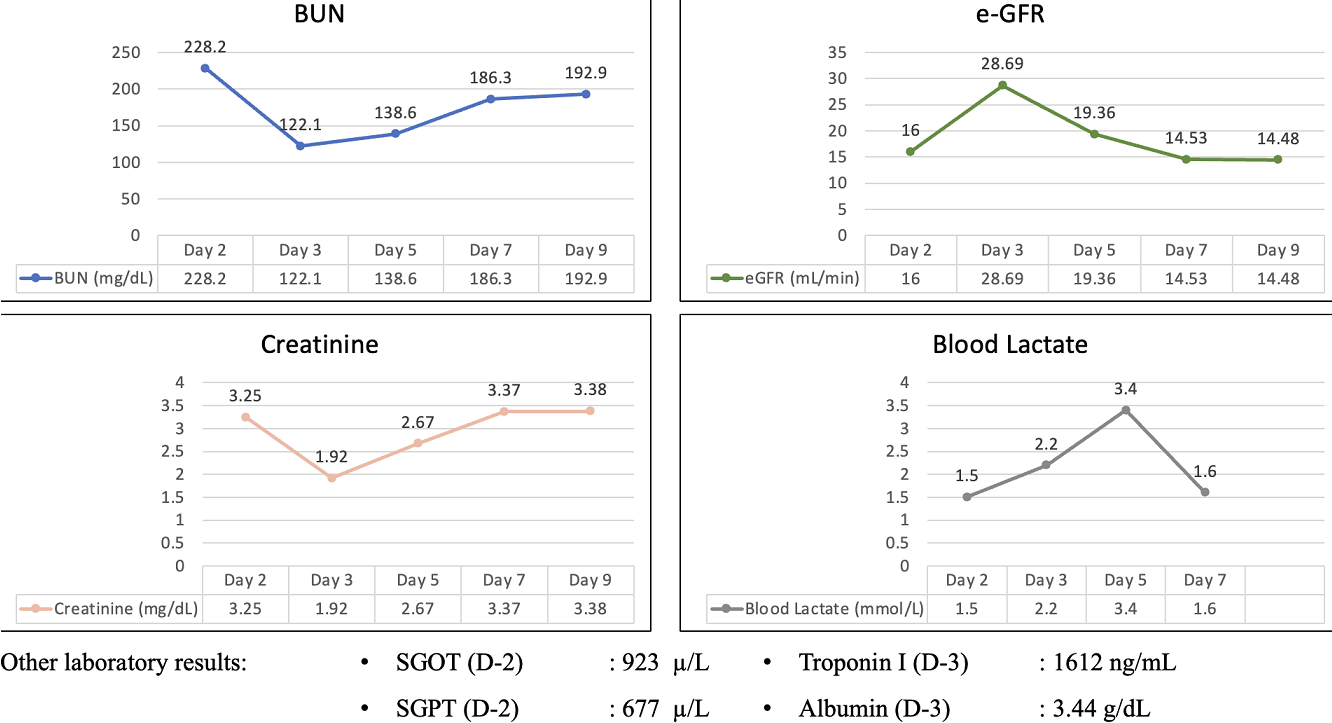
changes in BUN, e-GFR, creatinine, and blood lactate levels of the patient during ICU admission

CRRT was conducted for 48 hours and clinical improvement was observed. Brain perfusion parameters, such as improved consciousness, spontaneous eye-opening, and the ability to follow simple commands (GCS E2M4VETT with endotracheal tube) were accompanied by a blood pressure of 140/84 mmHg, irregular heart rate of 50 bpm, and bigeminy ventricular extrasystole. Kidney function improved with increased urine production (Figure 1), decreased serum creatinine level (to 1.92 mg/dL; Figure 2), and improved eGFR (to 19.36 mL/min; Figure 2). Pneumonia improved with the administration of a combination of antibiotics: meropenem 2g every 8h, levofloxacin 1g per day, and fluconazole 200 mg every 12h. An early tracheostomy was performed considering the longer weaning process from the ventilator in patients with heart failure (HF).

CRRT was discontinued due to filter saturation and supportive hemodialysis was initiated. The acute kidney injury score of the patient improved with a combination of HD and continuous diuretic therapy, achieving fluid balance.

**Follow-up and outcome of interventions:** on day 7 of treatment, weaning from mechanical ventilation was successful. The patient was transferred to the high-care unit (HCU) on day 10 of treatment, after three days without mechanical ventilation interventions. At this time, the patient showed good consciousness and a urine production of 1.4 mL/kg/h with minimal inotropic support.

**Patient perspective:** patients showed good consciousness and enough urine production after discharge from ICU.

**Informed consent:** It was obtained from the patient.

## Discussion

The US National Heart, Lung, and Blood Institute defines CRS as a condition resulting from the close connection between the cardiovascular system and the kidneys that leads to an increase in the amount of circulating blood, an exacerbation of HF, and the onset of kidney disease [4,5]. The initial proposed definition of CRS referred only to the impact of heart damage on pre-existing kidney failure; however, it is currently clear that the heart or kidneys can be the primary location of early injury. Hence, CRS can result from concurrent impairment of both the heart and kidneys, irrespective of the order in which they are affected [4].

According to the Acute Dialysis Quality Initiative (ADQI), CRS can be classified into five subcategories based on disease progression (Table 1). The criteria used to categorize these include the main location of the damage (heart or kidneys), other disorders affecting both organs and the type of condition (acute or chronic). CRS is characterized by heart dysfunction leading to kidney function impairment, whereas cardiorenal syndrome (a subcategory of CRS) is characterized by primary kidney failure that precipitates heart function impairment (Table 2) [4,5].

**Table 1:** five cardiorenal syndrome (CRS) subtypes (adapted from Rangaswami et al. [5])

Phenotype	Nomenclature	Description	Clinical Examples
Type 1 CRS	Acute CRS	Heart failure leading to acute kidney injury	Acute heart failure leading to acute kidney injury
			Acute coronary syndrome leading to acute kidney injury and cardiogenic shock
Type 2 CRS	Chronic CRS	Chronic heart failure leading to chronic kidney disease	Chronic heart failure
Type 3 CRS	Acute renocardiac syndrome	Acute kidney injury leading to acute heart failure	Heart failure in the setting of AKI from volume overload, inflammatory surge, and metabolic disturbances in uremia
Type 4 CRS	Chronic renocardiac syndrome	Chronic kidney disease leading to chronic heart failure	Heart failure and left ventricular hypertrophy from chronic kidney disease-associated cardiomyopathy
Type 5 CRS	Secondary CRS	Systemic process leading to kidney and heart failure	Cirrhosis, sepsis, and amyloidosis

**Table 2:** pathophysiology of CRS (adapted from Prastaro *et al*. [4])

Mechanisms	Mediators	Cardiac effects	Kidney effects
**Increased intra-abdominal and Central venous pressures**	Increased activation of SNS/RAAS	Remodeling heart	Decreased GFR
	Increased sodium and water retention	Chronic/acute HF	Renal venous congestion
**Decreased cardiac index and cardiac output**	Peripheral vasodilation	SNS/RAAS activation, detrimental cardiac function	Kidney ischemia
	Decreased perfusion pressure	Heart ischemia from diminished perfusion	Decreased kidney perfusion
	Decreased vascular resistance		
**Neurohormonal dysregulation: activation of RAAS, SNS, and AVP**	Oxidative stress	Hypertension	Inflammation accompanied by fibrotic impact
	Increased ET-1 expression	Inflammation accompanied by fibrotic impact	Increased sodium and water reabsorption
	Increased aldosterone, Ang II and renin secretion	Ventricular disfunction	Decreased GFR
	Impaired baroreceptor reflexes	Ventricular hypertrophy	Arteriolar vasoconstriction
**Oxidative stress**	Increased Uremic toxin-mediated cytokines release	Fibrosis and Inflammation	Interstitial fibrosis and inflammation
	Increased activity of NADPH oxidase	Endothelial dysfunction	Fibrosis
	Increased ROS production	Rapid progression of atherosclerosis	Rapid progression of atherosclerosis
		Ventricular hypertrophy	Endothelial dysfunction
**Inflammatory mediators**	Increased CRP, IL-6, IL-1 family, and TNF-alpha	Myocardial cells apoptosis	Glomerular injury caused by apoptosis of mesangial cells
		Hypertrophy and left ventricular dysfunction	Fibrosis and inflammation
		Fibrosis and inflammation	Atherosclerosis
		Rapid progression of atherosclerosis	
**Renal failure-related disturbances**	Coagulation imbalances	Arrythmias	Increased perivascular and interstitial fibrosis
	Electrolyte disturbances	Ischemia	Inflammation
	Acidemia	Hypertrophy and left ventricular dysfunction	Atherosclerosis
	Anemia (EPO resistance)	Vascular calcification and atherosclerosis	
	Ca-P and FGF23 abnormalities	Endothelial dysfunction	
	Oxidative stress		
	Increased chronic inflammatory cytokines		
	Increased PBUTs		
**Iatrogenic factors**	Contrast agents	Hypotension	Nephrotoxicity
	Drugs (diuretics, ARNI, ARBs, and ACE-I)		Decreased GFR

Note: SNS, sympathetic nervous system; ROS, reactive oxygen species; RAAS, renin-angiotensin-aldosterone system; CRP, C-reactive protein; PBUTs, protein-bound uremic toxins; HF, heart failure; GFR, glomerular filtration rate; FGF-23, fibroblast growth factor 23; ARNI, angiotensin receptor-neprilysin inhibitor; ARBs, angiotensin receptor blockers; IL-1, interleukin-1; Ang II, angiotensin II; ACEi, angiotensin-converting enzyme inhibitor; EPO, erythropoietin; ET-1, endothelin-1; NADPH, nicotinamide adenine dinucleotide phosphate; IL-6, interleukin-6; Ca-P, calcium-phosphate; TNF-alpha, tumor necrosis factor-alpha.

During acute episodes, CRS type 1 involves a reduction in cardiac function that results in renal function impairment. Renal impairment is present in 25% of patients diagnosed with ADHF. The incidence of CRS type 1 is 25.4%, with more than 30% of patients hospitalized for ADHF having a history of kidney insufficiency and 20% presenting with serum creatinine levels greater than 2.0 mg/dL [4].

**Figure 3:**
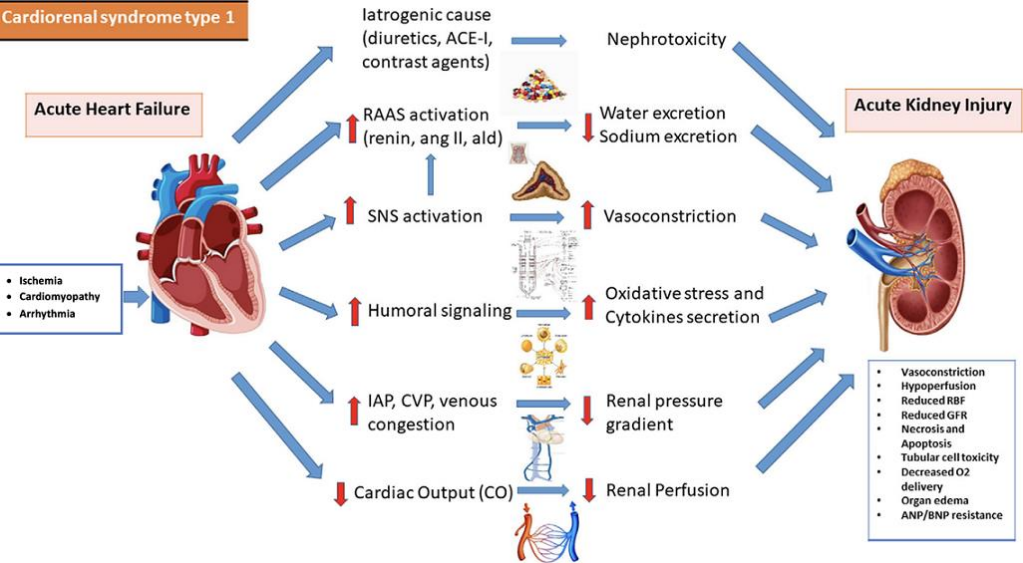
pathophysiology of CRS-Type 1 [4]

The patient in this case study had ADHF and an increase in serum creatinine level (3.25 mg/dL). The decline of kidney function was linked to renal hypoperfusion resulting from reduced cardiac output. ADHF resulted in both elevated central venous pressure (CVP) and volume overload. The congestion and elevated venous pressure diminished the blood flow rate within the glomerular capillary system, leading to sluggish intravascular flow, glomerular dysfunction, and reduced urine output. Increased CVP has also been directly linked to kidney function (Figure 3) [4].

The renin-angiotensin-aldosterone system (RAAS) contributes to the progression of kidney damage and the exacerbation of HF. In HF patients, this neurohormonal pathway is implicated in the restoration of tissue perfusion. Elevated renin levels can affect a rise in angiotensin II (Ang II), which can cause systemic detrimental impacts on the kidneys. Ang II induces efferent arteriolar vasoconstriction and increased renal plasma flow filtered via the glomerulus, leading to reduced hydrostatic pressure, enhanced elevated peritubular oncotic pressure, and salt reabsorption in the proximal tubule. Ang II also stimulates the synthesis of endothelin-1 (ET-1) in the kidneys and improves the aldosterone-stimulated reabsorption of sodium in the distal nephron. The peptide ET-1 is a profibrotic and proinflammatory agent, as well as a powerful vasoconstrictor that induces renal injury through pathological alterations. Moreover, the increase in oxidative stress involves activation of the sympathetic nervous system, stimulation of the RAAS, and volume expansion. In addition, nephrotoxic drugs such as certain antibiotics and nonsteroidal anti-inflammatory drugs administered to ADHF patients during inpatient hospital treatment can decrease kidney function [4].

The escalation of kidney failure can be attributed to its manifestation and possible etiology, as well as its correlation with diuretic response and functional condition. Complete decongestion should be achieved in patients who have positive diuretic responses, as residual congestion at hospital discharge is the primary predictor of readmission [2,6]. It is essential to ensure the optimal dosage is administered, provide a first evaluation of the diuretic effect by measuring urine volume and sodium excretion, and promptly increase the diuretic dosage when needed. The European Society of Cardiology HF guidelines (2021) recommend that vasodilators be used with ADHF patients (with systolic blood pressures more than 90 mmHg) who are hemodynamically stable. Patients with fluid overload and progressive acute kidney injury should generally be provided ultrafiltration as a final option [3]; however, early ultrafiltration may improve prognosis [2].

Hemodialysis (HD) is the removal of solutes through diffusion across a membrane from a region of high concentration to one of low concentration. During dialysis, a semi-permeable membrane separates the flow of blood from that of the dialysate, which is an electrolyte solution, and the two flow in opposite directions. Hemofiltration involves the pressure differential between the two sides of the dialysis membrane inducing the convection of water (ultrafiltration) and the release of tiny to medium-sized molecules. These molecules then flow through the more permeable membrane and are considered waste [7]. Dialysis is typically required when pharmaceutical therapies are no longer effective in controlling the hypervolemia or systemic toxification induced by renal excretion malfunction. However, in the context of CRS, dialysis is defined in a more comprehensive manner (conventional hemodialysis, peritoneal dialysis, extracorporeal ultrafiltration, continuous procedures) and may be utilized in cases with only moderate impairments. The primary condition for the use of dialysis is refractory hypervolemia (since diuretics are generally ineffective for this condition) [6].

In the case patient, HD was performed on day 2 of ICU treatment; however, the patient experienced arrhythmia during the dialysis process and HD was discontinued and replaced with CRRT. After 48 hours of CRRT, consciousness and kidney function improved, while creatinine level and urine production decreased. Increased mortality rates are directly proportional to the decline in urine output per hour or the rise in serum creatinine level [2]. Promptly implementing hemofiltration, effectively managing infective inflammation and fluid overload, and maintaining a stable cardiac and renal hemodynamic cycle can improve the prognosis [8].

Common indicators to favor the use of CRRT procedures include: 1) biochemical markers, such as urea and creatinine, which have different threshold values (many studies have confirmed that early or preventative use of CRRT significantly improves survival when compared to delayed CRRT treatment); 2) oliguria onset (one study revealed that initiating dialysis when the urine volume declined to below 30 mL/h resulted in a superior survival rate compared to initiation at volumes below 20 mL/h); 3) fluid volume status (one study indicated that a positive fluid balance was significantly linked to high mortality rates) [8].

CRRT plays a role in cytokine removal. Elevated levels of circulating cytokines (IL-6, and TNFα) are present in chronic HF cases, particularly those caused by cardiomyopathy, and these inflammatory signaling mediators can lead to dilation and hypertrophy and contribute to poor prognosis and the advancement of left ventricular dysfunction. Continuous veno-venous hemofiltration (CVVH) can enhance the survival of patients with cardiomyopathy, possibly due to the non-specific elimination of extracorporeal cytokines, resulting in longer duration of each treatment and smaller ultrafiltration rates [2]. In addition, ultrafiltration removes a greater amount of sodium than diuretic therapy using the same amount of water and produces more accurate fluid target determinations [7].

## Conclusion

Patients with ADHF treated in the hospital may develop CRS, which can increase mortality. The use of CRRT can be a therapeutic option for patients with CRS. Through ultrafiltration, CRRT is capable of removing circulating cytokines in the blood, reducing volume overload, and addressing electrolyte imbalance, thereby enhancing the function of the heart and kidneys and potentially improving prognoses.
